# Hot water immersion as a potential substitute for strength training during the COVID-19 pandemic

**DOI:** 10.3389/fphys.2023.1103609

**Published:** 2023-02-09

**Authors:** Metodija Kjertakov, Shavin Chandrasiri, Aaron Petersen

**Affiliations:** Institute for Health and Sport, Victoria University, Melbourne, VIC, Australia

**Keywords:** muscle strength, muscle mass, COVID-19, passive heat exposure, hot bath treatment

## Introduction

Muscle strength and mass can impact the course of the disease in patients with coronavirus disease-2019 (COVID-19) (Grigioni et al., 2023; Kara et al., 2021; Pucci et al., 2022). Kara et al. (2021) measured handgrip strength at hospital admission of 312 COVID-19 patients and reported that patients with low handgrip strength had three times higher risk for developing pneumonia compared to those with high handgrip strength. The study by Pucci et al. (2022), conducted on 118 hospitalised COVID-19 patients, found that handgrip strength at admission to the hospital was 26% lower in patients who later needed endotracheal intubation versus those who did not. Grigioni et al. (2023) used computed tomography to measure skeletal muscle mass at the thoracic level in 244 hospitalised COVID-19 patients within 48 h of their hospital admission and reported that those with low muscle mass had longer hospital stay (25 days vs. 13 days) than their counterparts with normal muscle mass. Collectively, the cited studies suggest that increasing muscle strength and mass *via* strength training during the COVID-19 pandemic could be an effective prophylactic strategy to strengthen resilience to severe forms of the disease in case a person gets infected by severe acute respiratory syndrome coronavirus 2. While guidelines for strength training during the COVID-19 pandemic have been published (Gentil et al., 2021; Gentil et al., 2022; Rodríguez et al., 2020; Sá Filho et al., 2020), they failed to provide alternative strategies for those unable or unwilling to exercise. Clearly, there is a need to introduce a non-exercise intervention aimed at increasing muscle strength and mass in the COVID-19 literature. The current paper provides evidence that passive heat exposure could be an alternative to strength training in increasing muscle strength and mass and proposes a home-based heating treatment.

### Evidence that passive heating can increase muscle strength and mass

The ability of chronic passive heat exposure to increase muscle strength in humans has been demonstrated in several studies (Goto et al., 2011; Kim et al., 2020b; Racinais et al., 2017). Maximal isometric force during knee extension increased by 5.8% following repeated heating of the thigh with a heat pad, so that vastus lateralis muscle temperature reached 38.3°C, for 8 h per day, 4 days per week, for 10 weeks (Goto et al., 2011). Thigh heating *via* a water-perfused garment for 90 min, 5 days per week for 8 weeks increased knee extension maximal isokinetic torque at 180°/s^−1^ by 6 and 5% after 4 and 8 weeks, respectively (Kim et al., 2020b). In contrast to those two studies, Labidi et al. (2021) found no change in the contractile properties of the gastrocnemius muscle following a 6-week intervention consisting of a daily increase in gastrocnemius muscle temperature to 37.6°C by applying heat pads for 8 h, 5 days per week. It is likely that the muscle temperature attained during the intervention in Labidi’s study was not of sufficient magnitude to induce beneficial changes in skeletal muscle. Interestingly, Racinais et al. (2017) observed improved muscle contractility (evidenced by increased electrically evoked peak twitch force of the soleus by 9% and maximal voluntary torque during plantar flexion by 17%) after only eleven daily 1-h heat exposures in a 48°C–50°C heat chamber where the participants’ core temperature was maintained at ∼39°C. Muscle temperature was not measured, but elsewhere, the same whole-body heating treatment was reported to increase muscle temperature to 38.8°C (Ihsan et al., 2020).

There is also evidence that passive heat exposure can increase muscle mass. In rodents, hypertrophy has been observed following immersion of the lower body in 39°C water for 30 min each day for 3 weeks (Kim et al., 2019) and following a single heat exposure in a heat chamber at 41°C for 60 min (Ohno et al., 2015). In humans, repeated heating of the thigh with a heat pad, as described above, increased rectus femoris and vastus lateralis muscle cross-sectional areas by 6.1 and 2.7%, respectively (Goto et al., 2011). Sauna bathing, which comprised five sets of 10 min exposures to 100°C (20%–30% relative humidity) with 5 min recovery at 22°C between sets, performed 3 days per week for 4 weeks, had no effect on whole body muscle mass but increased muscle mass of the right leg when measured 2 weeks after the last sauna session (Toro et al., 2021). Heat exposure may also hasten muscle re-growth following a period of disuse. For example, in rats which were re-ambulated following 7 days of immobilization, soleus muscle regrowth was 20% greater in animals that had their rectal temperature raised to 41°C–41.5°C for 30 min every second day for 7 days compared to non-heated animals (Selsby et al., 2007). Given that severe forms of COVID-19 are associated with muscle wasting (Attaway et al., 2022), the findings of Selsby et al. (2007) are highly relevant in the context of rehabilitation of those COVID-19 survivors who cannot exercise.

### Potential mechanisms behind increased muscle strength and mass with passive heating

Our current understanding of the molecular mechanisms underpinning the ergogenic effects of chronic passive heat exposure on muscle strength and mass is based only on a handful of mechanistic studies. In one of the earliest studies in this area, Kobayashi et al. (2005) analysed skeletal muscle tissue of rats that were exposed to environmental heat stress (41°C) in a heat chamber for 60 min and found increased expression of molecules (heat shock protein 72 and calcineurin) that are considered to participate in the process of muscle hypertrophy. The latter notion is based on observations that inhibition of calcineurin expression (Dunn et al., 1999) and absence of heat shock transcription factor 1 (Ohno et al., 2015) impairs muscle hypertrophy in response to hypertrophic stimuli. Another molecular mechanism by which heat exposure could induce hypertrophy is stimulating the mammalian target rapamycin (mTOR) signaling pathway, a key regulator of muscle protein synthesis (Yoon, 2017). Indeed, Yoshihara et al. (2013) demonstrated that a rise in the temperature of rat skeletal muscle to 41°C induced by hot water immersion increases phosphorylation of the mTOR signaling components protein kinase B and 70-kDa ribosomal protein S6, which is indicative of increased mTOR activity. The findings of Yoshihara et al. (2013) were subsequently confirmed by Tamura et al. (2014) in a study that had rats receiving whole-body heating treatment in a heat chamber (40°C) for 30 min. In a more recent study in a human model, Ihsan et al. (2020) also observed activation of the mTOR pathway in the *vastus lateralis* in response to heat stress applied by having the participants resting in a heat chamber at 44°C–50°C for 60 min where their core and muscle temperatures increased to 39.1°C and 38.8°C, respectively.

The findings of Ihsan et al. (2020), Kobayashi et al. (2005), Tamura et al. (2014), and Yoshihara et al. (2013) point out the possible molecular mechanisms behind heat-induced hypertrophy in the studies (Goto et al., 2011; Kim et al., 2019) cited in the previous section. Furthermore, in the latter two studies, increased hypertrophy helps to explain the reported increase in muscle strength (Folland and Williams., 2007). However, the fact that the heating treatment in the study by Kim et al. (2020b) led to increased muscle strength despite no significant change in muscle mass suggests that adaptations other than hypertrophy could also contribute to enhanced force development following chronic heat exposure. Increased muscle contractility following a single heat exposure has been attributed to increased calcium kinetics and increased intramuscular fluid (Rodrigues et al., 2022), but whether these physiological events played a role in the increased muscle strength in Kim’s study remains unknown.

### Home-based heating treatment

While researchers have access to various heating methods in laboratory settings, the application of heating treatment in home conditions for many potential users would only be possible *via* hot baths (Kjertakov and Petersen, 2022). Head-out submersion in ∼40°C water is the most described hot water immersion method in the passive heating literature (Gerrett et al., 2021; Greenfield et al., 2021; Hashiguchi et al., 2002; Lee et al., 2012; Lovell et al., 2008; Naumann et al., 2017), which typically induces a magnitude of hyperthermia (≥39°C) similar to that in the studies by Ihsan et al. (2020) and Racinais et al. (2017) previously cited. However, the 40°C water immersion treatment may not be the best alternative to the heating methods used in the latter two studies mainly because it usually causes unbearable discomfort after only 30–40 min from the onset of immersion with a resultant termination of the treatment (Greenfield et al., 2021 ; Naumann et al., 2017). Recall that the heating treatments used by Ihsan et al. (2020) and Racinais et al. (2017) lasted 60 min. Immersion in 39°C water extends the length of the treatment to 60 min but the thermal response (end-treatment core temperature of 38.6°C) to this water temperature (Leicht et al., 2019) is lower than that reported by Ihsan et al. (2020) and Racinais et al. (2017). Since it is currently unknown what the minimal “dose” of heat stress is to induce the desired adaptations in human skeletal muscle (Kim et al., 2020a), we use the studies of Ihsan et al. (2020) and Racinais et al. (2017) as references to recommend a hot water immersion treatment aimed at potentially increasing both muscle strength and mass. Accordingly, we propose a 60 min hot water treatment where the temperature of the water for the first 20 min will be maintained at 40°C, and then it will be reduced to 39°C for the rest of the treatment. This idea is based on the observation that it takes 20 min for the core temperature to get close to 39°C in response to head-out immersion in 40°C water (Lovell et al., 2008), and on the assumption that the core temperature will equilibrate with the water temperature during the next 40 min of the treatment. Effective implementation of this treatment calls for continuous monitoring of the water temperature throughout the treatment so one knows when to allow some water to drain and add the same volume of hot water into the bath in order to maintain the correct immersion temperature and depth. The hot water immersion protocol we are proposing is less aggressive compared to the existing 40°C water immersion protocol, and as such it is expected to allow one to remain submerged for 60 min while experiencing thermal stress similar to that reported by Ihsan et al. (2020) and Racinais et al. (2017). Furthermore, the risk of heat stroke associated with immersion in 40°C water (Lee et al., 2010) is reduced with the proposed protocol because this protocol is expected to limit the increase in core temperature to ∼39°C. This value of core temperature is lower than that (>40°C) observed after the onset of heat stroke (Epstein and Yanovich, 2019; Kjertakov and Epstein, 2013). Nevertheless, the treatment should be terminated in the case of an onset of nausea, dizziness, or unbearable discomfort. It also needs to be mentioned that the risk of transient symptomatic hypotension associated with the 40°C water immersion treatment (Naumann et al., 2017) also applies to our protocol. Therefore, the potential users of our treatment should not hurry to stand after completing it and should sit for a few minutes after getting out of the bath to allow blood pressure to return to normal and avoid the potential risk of falling due to syncope (James et al., 2021).

## Conclusion

The hot water immersion treatment proposed in this paper is expected to replicate the characteristics of the heating treatments used by Ihsan et al. (2020) and Racinais et al. (2017) in relation to their duration and the increase in core and muscle temperatures they induce, and as a result to stimulate similar beneficial responses in skeletal muscle as in those two studies. The proposed treatment is also expected to be beneficial in the context of rehabilitation of COVID-19 patients who have experienced significant muscle wasting over the course of the disease. Based on the human studies demonstrating the positive effects of passive heating on muscle strength and mass, using the proposed treatment for 5–7 days a week should induce the desired adaptations in skeletal muscle.

## References

[B1] Attaway A. Welch N. Dasarathy D. Amaya‐Hughley J. Bellar A. Biehl M. (2022). Acute skeletal muscle loss in SARS‐CoV‐2 infection contributes to poor clinical outcomes in COVID‐19 patients. J. Cachexia Sarcopenia Muscle 13 (5), 2436–2446. 10.1002/jcsm.13052 35851995 PMC9350025

[B2] Dunn S. E. Burns J. L. Michel R. N. (1999). Calcineurin is required for skeletal muscle hypertrophy. J. Biol. Chem. 274 (31), 21908–21912. 10.1074/jbc.274.31.21908 10419511

[B3] Epstein Y. Yanovich R. (2019). Heatstroke. N. Engl. J. Med. 380 (25), 2449–2459. 10.1056/NEJMra1810762 31216400

[B4] Fennel Z. J. Amorim F. T. Deyhle M. R. Hafen P. S. Mermier C. M. (2022). The heat shock connection: Skeletal muscle hypertrophy and atrophy. Am. J. Physiol. Regul. Integr. Comp. Physiol. 323 (1), R133–R148. 10.1152/ajpregu.00048.2022 35536704

[B5] Folland J. P. Williams A. G. (2007). The adaptations to strength training: Morphological and neurological contributions to increased strength. Sports Med. 37 (2), 145–168. 10.2165/00007256-200737020-00004 17241104

[B6] Gentil P. De Lira C. A. B. Coswig V. Barroso W. K. S. Vitorino P. V. D. O. Ramirez-Campillo R. (2021). Practical recommendations relevant to the use of resistance training for COVID-19 survivors. Front. Physiol. 12, 142. 10.3389/fphys.2021.637590 PMC7966515 33746777

[B7] Gentil P. de Lira C. A. B. Vieira C. A. Ramirez-Campillo R. Haghighi A. H. Clemente F. M. (2022). Resistance training before, during, and after COVID-19 infection: What have we learned so far? Int. J. Environ. Res. Public Health 19 (10), 6323. 10.3390/ijerph19106323 35627861 PMC9141848

[B36] Gerrett N. Alkemade P. Daanen H. (2021). Heat reacclimation using exercise or hot water immersion. Med. Sci. Sports Exerc. 53 (7), 1517–1528. 10.1249/MSS.0000000000002612 34127636 PMC8208095

[B8] Goto K. Oda H. Kondo H. Igaki M. Suzuki A. Tsuchiya S. (2011). Responses of muscle mass, strength and gene transcripts to long-term heat stress in healthy human subjects. Eur. J. Appl. Physiol. 111 (1), 17–27. 10.1007/s00421-010-1617-1 20803152

[B9] Greenfield A. M. Pereira F. G. Boyer W. R. Apkarian M. R. Kuennen M. R. Gillum T. L. (2021). Short-term hot water immersion results in substantial thermal strain and partial heat acclimation; comparisons with heat-exercise exposures. J. Therm. Biol. 97, 102898. 10.1016/j.jtherbio.2021.102898 33863451

[B10] Grigioni S. Lvovschi V. E. Tamion F. Joly L. M. Coëffier M. Van Elslande H. (2023). Low thoracic skeletal muscle index is associated with negative outcomes in 244 patients with respiratory COVID-19. Clin. Nutr. 42 (2), 102–107. 10.1016/j.clnu.2022.11.011 36521254 PMC9674398

[B37] Hashiguchi N. Ni F. Tochihara Y. (2002). Effects of room temperature on physiological and subjective responses during whole-body bathing, half-body bathing and showering. J. Physiol. Anthropol. Appl. Human. Sci. 21 (6), 277–283. 10.2114/jpa.21.277 12612399

[B11] Ihsan M. Deldicque L. Molphy J. Britto F. Cherif A. Racinais S. (2020). Skeletal muscle signaling following whole-body and localized heat exposure in humans. Front. Physiol. 839, 839. 10.3389/fphys.2020.00839 PMC7381176 32765299

[B12] James T. J. Corbett J. Cummings M. Allard S. Young J. S. Towse J. (2021). Timing of acute passive heating on glucose tolerance and blood pressure in people with type 2 diabetes: A randomized, balanced crossover, control trial. J. Appl. Physiol. 130 (4), 1093–1105. 10.1152/japplphysiol.00747.2020 33411640

[B13] Kara Ö. Kara M. Akın M. E. Özçakar L. (2021). Grip strength as a predictor of disease severity in hospitalized COVID-19 patients. Heart Lung 50 (6), 743–747. 10.1016/j.hrtlng.2021.06.005 34217985 PMC8192888

[B14] Kim K. Reid B. A. Ro B. Casey C. A. Song Q. Kuang S. (2019). Heat therapy improves soleus muscle force in a model of ischemia-induced muscle damage. J. Appl. Physiol. 127 (1), 215–228. 10.1152/japplphysiol.00115.2019 31161885

[B15] Kim K. Monroe J. C. Gavin T. P. Roseguini B. T. (2020a). Skeletal muscle adaptations to heat therapy. J. Appl. Physiol. 128 (6), 1635–1642. 10.1152/japplphysiol.00061.2020 32352340 PMC7311689

[B16] Kim K. Reid B. A. Casey C. A. Bender B. E. Ro B. Song Q. (2020b). Effects of repeated local heat therapy on skeletal muscle structure and function in humans. J. Appl. Physiol. 128 (3), 483–492. 10.1152/japplphysiol.00701.2019 31971474

[B17] Kjertakov M. Epstein Y. (2013). Exertional heat stroke in athletes. Mace J. Med. Sci. 1 (1), 473–477. 10.3889/mjms.1857-5773.2013.0308

[B18] Kjertakov M. Petersen A. (2022). Hot water immersion could be an effective alternative to physical exercise in improving cardiovascular fitness during the COVID-19 pandemic. Front. Physiol. 2381. 10.3389/fphys.2022.1035183 PMC9732457 36505081

[B19] Kobayashi T. Goto K. Kojima A. Akema T. Uehara K. Aoki H. (2005). Possible role of calcineurin in heating-related increase of rat muscle mass. Biochem. Biophys. Res. Commun. 331 (4), 1301–1309. 10.1016/j.bbrc.2005.04.096 15883017

[B20] Labidi M. Ihsan M. Behan F. P. Alhammoud M. Smith T. Mohamed M. (2021). Six weeks of localized heat therapy does not affect muscle mass, strength and contractile properties in healthy active humans. Eur. J. Appl. Physiol. 121 (2), 573–582. 10.1007/s00421-020-0454-9 33159573

[B38] Lee S. Ishibashi S. Shimomura Y. Katsuura T. (2012). Physiological functions of the effects of the different bathing method on recovery from local muscle fatigue. J. Physiol. Anthropol. 31 (1), 26. 10.1186/1880-6805-31-26 22980588 PMC3576248

[B21] Lee C. W. Perng C. L. Huang Y. S. Luo J. C. Hung C. L. Lin H. C. (2010). Multiple organ failure caused by non-exertional heat stroke after bathing in a hot spring. J. Chin. Med. Assoc. 73 (4), 212–215. 10.1016/S1726-4901(10)70044-7 20457444

[B22] Leicht C. A. James L. J. Briscoe J. H. Hoekstra S. P. (2019). Hot water immersion acutely increases postprandial glucose concentrations. Physiol. Rep. 7 (20), e14223. 10.14814/phy2.14223 31642205 PMC6805849

[B23] Lovell R. Madden L. McNaughton L. R. Carroll S. (2008). Effects of active and passive hyperthermia on heat shock protein 70 (HSP70). Amino Acids 34 (2), 203–211. 10.1007/s00726-007-0507-2 17928942

[B24] Naumann J. Grebe J. Kaifel S. Weinert T. Sadaghiani C. Huber R. (2017). Effects of hyperthermic baths on depression, sleep and heart rate variability in patients with depressive disorder: A randomized clinical pilot trial. BMC Complement. Altern. Med. 17 (1), 172–179. 10.1186/s12906-017-1676-5 28351399 PMC5371197

[B25] Ohno Y. Egawa T. Yokoyama S. Nakai A. Sugiura T. Ohira Y. (2015). Deficiency of heat shock transcription factor 1 suppresses heat stress‐associated increase in slow soleus muscle mass of mice. Acta Physiol. 215 (4), 191–203. 10.1111/apha.12600 26347147

[B26] Pucci G. D’Abbondanza M. Curcio R. Alcidi R. Campanella T. Chiatti L. (2022). Handgrip strength is associated with adverse outcomes in patients hospitalized for COVID-19-associated pneumonia. Intern Emerg. Med. 17 (7), 1997–2004. 10.1007/s11739-022-03060-3 35930184 PMC9362345

[B27] Racinais S. Wilson M. G. Périard J. D. (2017). Passive heat acclimation improves skeletal muscle contractility in humans. Am. J. Physiol. Regul. Integr. Comp. Physiol. 312 (1), R101–R107. 10.1152/ajpregu.00431.2016 27903515

[B28] Rodrigues P. Trajano G. S. Stewart I. B. Minett G. M. (2022). Potential role of passively increased muscle temperature on contractile function. Eur. J. Appl. Physiol. 122 (10), 2153–2162. 10.1007/s00421-022-04991-7 35771296 PMC9463203

[B29] Rodríguez M. Á. Crespo I. Olmedillas H. (2020). Exercising in times of COVID-19: What do experts recommend doing within four walls? Rev. Esp. Cardiol. Engl. Ed. 73 (7), 527–529. 10.1016/j.recesp.2020.04.002 32414660 PMC7142674

[B30] Sá Filho A. S. Miranda T. G. de Paula C. C. Barsanulfo S. R. Teixeira D. Monteiro D. (2020). COVID-19 and quarantine: Expanding understanding of how to stay physically active at home. Front. Psychol. 11, 566032. 10.3389/fpsyg.2020.566032 33192841 PMC7658189

[B31] Selsby J. T. Rother S. Tsuda S. Pracash O. Quindry J. Dodd S. L. (2007). Intermittent hyperthermia enhances skeletal muscle regrowth and attenuates oxidative damage following reloading. J. Appl. Physiol. 102 (4), 1702–1707. 10.1152/japplphysiol.00722.2006 17110516

[B32] Tamura Y. Matsunaga Y. Masuda H. Takahashi Y. Takahashi Y. Terada S. (2014). Postexercise whole body heat stress additively enhances endurance training-induced mitochondrial adaptations in mouse skeletal muscle. Am. J. Physiol. Regul. Integr. Comp. Physiol. 307 (7), R931–R943. 10.1152/ajpregu.00525.2013 25080501

[B33] Toro V. Siquier-Coll J. Bartolomé I. Pérez-Quintero M. Raimundo A. Muñoz D. (2021). Effects of twelve sessions of high-temperature sauna baths on body composition in healthy young men. Int. J. Environ. Res. Public Health 18 (9), 4458. 10.3390/ijerph18094458 33922289 PMC8122786

[B34] Yoon M. S. (2017). mTOR as a key regulator in maintaining skeletal muscle mass. Front. Physiol. 8, 788. 10.3389/fphys.2017.007 29089899 PMC5650960

[B35] Yoshihara T. Naito H. Kakigi R. Ichinoseki‐Sekine N. Ogura Y. Sugiura T. (2013). Heat stress activates the Akt/m TOR signalling pathway in rat skeletal muscle. Acta Physiol. 207 (2), 416–426. 10.1111/apha.12040 23167446

